# Efficacy of pediatric integrative manual therapy in positional plagiocephaly: a randomized controlled trial

**DOI:** 10.1186/s13052-021-01079-4

**Published:** 2021-06-05

**Authors:** Iñaki Pastor-Pons, María Orosia Lucha-López, Marta Barrau-Lalmolda, Iñaki Rodes-Pastor, Ángel Luis Rodríguez-Fernández, César Hidalgo-García, Jose Miguel Tricás-Moreno

**Affiliations:** 1 Departamento de Fisiatría y Enfermería, Unidad de Investigación en Fisioterapia, Facultad de Ciencias de la Salud, Universidad de Zaragoza, Domingo Miral, s/n, 50009 Zaragoza, Spain; 2 Instituto de Terapias Integrativas, Constitución 29 Dplo, 50001 Zaragoza, Spain; 3 Departamento de Fisioterapia, Facultad de Medicina, Universidad San Pablo CEU, Urbanización Montepríncipe, 28925, Alcorcón, Madrid, Spain

**Keywords:** Positional Plagiocephaly, Deformational Plagiocephaly, Manual therapy, Physical therapy

## Abstract

**Background:**

Positional plagiocephaly frequently affects healthy babies. It is hypothesized that manual therapy tailored to pediatrics is more effective in improving plagiocephalic cranial asymmetry than just repositioning and sensory and motor stimulation.

**Methods:**

Thirty-four neurologically healthy subjects aged less than 28 weeks old with a difference of at least 5 mm between cranial diagonal diameters were randomly distributed into 2 groups. For 10 weeks, the pediatric integrative manual therapy (PIMT) group received manual therapy plus a caregiver education program, while the controls received the same education program exclusively. Cranial shape was evaluated using anthropometry; cranial index (CI) and cranial vault asymmetry index (CVAI) were calculated. Parental perception of change was assessed using a visual analogue scale (− 10 cm to + 10 cm).

**Results:**

CVAI presented a greater decrease in PIMT group: 3.72 ± 1.40% compared with 0.34 ± 1.72% in the control group (*p* = 0.000). CI did not present significant differences between groups. Manual therapy led to a more positive parental perception of cranial changes (manual therapy: 6.66 ± 2.07 cm; control: 4.25 ± 2.31 cm; *p* = 0.004).

**Conclusion:**

Manual therapy plus a caregiver education program improved CVAI and led to parental satisfaction more effectively than solely a caregiver education program.

**Trial registration:**

Trial registration number: NCT03659032; registration date: September 1, 2018. Retrospectively registered.

## Background

Head and neck asymmetries are very common in typical healthy newborns [1]. Within these asymmetries, positional plagiocephaly (PP) is a general term describing cranial distortion from pre- or postnatal forces on the infant head [2, 3]. PP features are asymmetrical occipital flattening, accompanied by anterior displacement of the ear on the same side, parietal protuberance on the opposite side, and often ipsilateral frontal protuberance, with fellow frontal flattening. These characteristics make the head look like a parallelogram when viewed from above [4]. Facial findings can be associated with the condition, but PP does not imply or connote this [5].

Prevalence data are limited and depend on the geographic location. However, the prevalence seems to be high as the best estimations of the presence of PP in infants range from 20 to 40% [6–8].

Many intrinsic and extrinsic factors can play a role before, during and after childbirth. Besides being associated with lying supine, the development of plagiocephaly is linked to gestational diabetes [9], male sex [10, 11], maternal age [12], skull circumference [12], prematurity [13], primiparity [10, 11], brachiocephaly [9, 10], intra-uterine constraints [14], prolonged labor [14], multiple births [14], improper fetal position during birth [14], use of obstetrical forceps or a suction cup [15], lengthy hospital stay [16], congenital torticollis [6, 17], head positional preference [10–12, 18], infant being awake in a prone position less than 3 times a day [10] and delayed motor milestone acquisition [10].

Although many cases of PP improve over time, scientific evidence suggests that conservative management strategies can safely and effectively minimize the degree of cranial asymmetry [19]. The controlled clinical trial carried out by Van Vlimmeren et al. (2008) is one of the highest quality studies. Those researchers compared an intervention group receiving standardized repositioning and physiotherapy treatment with a control group that received the usual care (parents received a leaflet describing basic preventive measures without further education or instructions to intervene). After the intervention, the ratio of babies with severe PP was significantly lower in the treatment group than in the control group. Their findings suggest that, without intervention, some babies with PP and positional preference could develop severe PP [20]. The results also imply an optimal time framework for the treatment, in which the earlier the intervention, the better the outcomes [19].

The main conservative treatment options for PP are parental education [21–27], repositioning [23–25, 28–30], physiotherapy [20, 28, 31–33] and orthotic helmet therapy [26, 29, 34–36].

Only a few studies have analyzed the effect of manual therapy on non-synostosis plagiocephaly [37–39]. Most of these studies have been unable to establish a sufficient level of evidence because of the general lack of proper samples sizes, control groups, or randomization.

The objective of this study was to analyze the effectiveness of adding a pediatric manual therapy approach to a caregiver education program in anthropometric cranial measurements and the subjective parental perception of the cranial shape change in infants with PP.

## Methods

The Ethics Committee at the Aragon Health Sciences Institute approved recruiting a cohort for this study (Registry No. C.P. - C.I. PI16/0275. Date: October 25, 2017). The study is registered at clinicaltrials.gov, with identification number NCT03659032. Registration date: September 1, 2018.

### Subjects

Pediatricians in Section III of the Aragon Health Services referred 34 subjects aged less than 28 weeks having signs of PP. The inclusion criterion was infants with a difference of at least 5 mm between cranial diagonal diameters [40], that is, infants with moderate or severe PP [41]. We excluded infants who had received orthotic treatment, physiotherapy or presented genetic, communicable, metabolic or neurological illness or craniosynostosis.

For the calculation of the sample size, we used non-published data from a previous pilot study with 41 subjects with similar characteristics and who had received a similar manual therapy approach as this present study. This pilot study obtained a decrease of 4,52 ± 2,91% in the cranial vault asymmetry index. The sample size was calculated using the GRANMO calculator (https://www.imim.cat/ofertadeserveis/software-public/granmo/), with the selection of two independent population means, bilateral contrast, with a α risk of 0.05, a ß risk of 0.20 and a ratio of 1 of the number of subjects between the groups. A minimal number of 7 subjects per group was obtained.

Subjects were randomized into 2 groups with a final number of 17 subjects per group. Subjects were randomized following a design generated with the on-line computer application at www.random.org/sequences. The evaluators were blinded to this design.

An informative document about the study was provided to the parents and an informed consent was signed after they had read the document and their questions about the study had been answered. Regulations and guidelines regarding freedom, absence of coercion, disclosure of economic interests, understandable and complete information, confidentiality and acceptance were followed [42].

### Measured parameters

Clinical and demographic data were extracted from the medical history and the testimony of the parents: age (weeks), birth weight (gr), sex, prematurity, instrumental delivery, firstborn, multiple birth, head positional preference, pediatrician diagnosis of congenital torticollis, plagiocephaly side, transport type and time that the infant spent in prone position awake and watched at 1 month (min) and at 2 months (min).

The following anthropometric parameters, constituting the dependent study variables, were measured: maximal cranial circumference (MCC) [43], cranial length, cranial width and diagonal cranial diameter taken from the frontozygomatic suture (fz) to the fellow lambdoid suture (lb) [44]. Interrater and intrarater reliability of cranial anthropometric measurements has been previously published [45]. From these data, the next qualifiers were calculated.

Cranial index (CI). The CI was calculated with the formula: “Cranial Width (mm)/Cranial Length (mm) ×100” [24]. Normal range described for CI is between 75 and 85% [44].

Cranial vault asymmetry (CVA). The CVA was calculated with the formula: “Long diagonal cranial diameter (mm) – Short diagonal cranial diameter (mm)” [46]. According to Mortenson & Steinbok, the CVA can be classified into the following categories: normal CVA < 3 mm, mild / moderate CVA ≤ 12 mm, moderate / severe CVA > 12 mm [41].

Cranial vault asymmetry index (CVAI). The CVAI was calculated using the formula “[Long diagonal cranial diameter (mm) – Short diagonal cranial diameter (mm)]/Short diagonal cranial diameter×100” [24]. The classification of the plagiocephaly severity scale (Children’s Healthcare of Atlanta, 2015) [47] is based on the CVAI and it describes the following levels: level 1: < 3.5%; level 2: 3.5 to 6.25%; level 3: 6.25 to 8.75%; level 4: 8.75 to 11.0%; level 5:> 11.0%. CVAI was established as the primary outcome measure.

At the end of the study, the parents were given a visual analogue scale (VAS) to evaluate their perception of head shape change [48]. The parents made a vertical mark on a line graduated from − 10 (much worse) to + 10 (much better) with an intermediate Item 0 (no change).

### Intervention

**Fig. 1:**
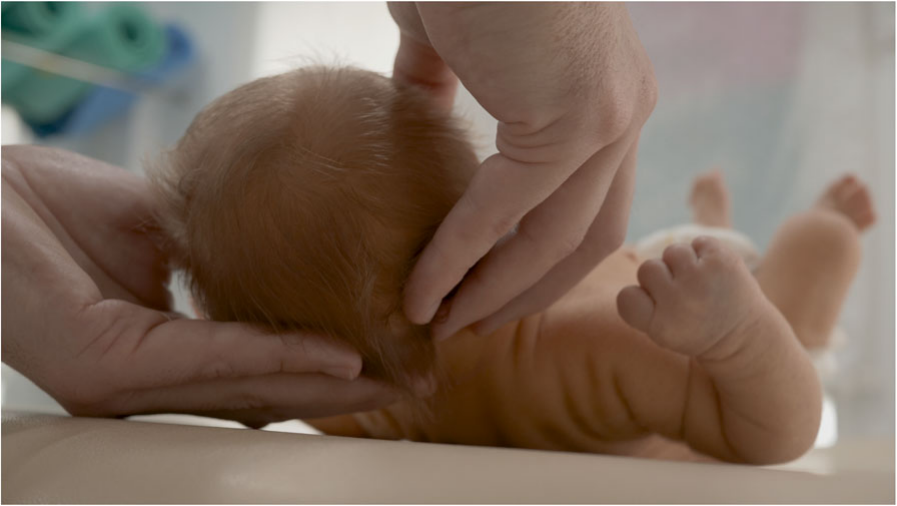
Cranial Base Molding Technique applied in the ;intervention group

Seventeen subjects received 10 sessions of manual therapy and a caregiver education program, an integrative concept of treatment that will be identified in the manuscript as pediatric integrative manual therapy (PIMT). Each PIMT treatment for remodeling the cranial deformation consisted of:

- one maneuver to mobilize the neuromeningeal tissue at the lumbosacral level, based on the technique of John E. Upledger [49] but adapted to the pediatric field. Very light traction is induced through the pelvis to stimulate a tissue response. The physiotherapist follows the movements of the baby’s pelvis according to the active movements, trying to move towards increasingly flexed positions.
- one technique for the cervical spine based on the works of Giammatteo [50]. Very slight traction is applied through the head and the active movement of the head is accompanied to different positions of flexion and extension, lateralization and rotation, stabilizing the atlas gently in a dorsal direction.
- one technique applying manual pressure to mold the skull base in the opposite direction from the PP torsion on the skull base, based on the work of Arbuckle [51] . The manual pressure was applied to the occipital bone to displace it dorsally, insisting on the flattest area (Fig. 1).
- two techniques; one to balance the intracranial membranous tension and one molding technique for decompressing coronal suture based on the work of Carreiro [52].

This PIMT protocol was applied by several pediatric physical therapists with specialized training and 4 years of experience. The effects of this PIMT treatment in the cervical spine mobility are described in another manuscript previously published [53].

Each manual therapy session was performed once a week with a duration of 20 min.

The caregiver education program consisted of a series of literature-based recommendations [54, 55] that encompassed repositioning, sensory and motor stimulation of the opposite side to the preferred one and prone positions. Parents were instructed with the help of a trained pediatric physiotherapist and an informative booklet about basic recommendations.

The 17 subjects from the control group received solely the same caregiver education program. The control group was convened once during the 10 weeks to monitor the process, listen to their difficulties, resolve their questions and insist on the importance of performing stimulation and repositioning.

### Statistical analyses

The Kolmogorov-Smirnov test with the Lilliefors correction was used to test the normality of the distribution of the quantitative variables; the Shapiro-Wilk test was used for this purpose if *n* < 30. A descriptive analysis of the qualitative variables was carried out, offering the percentages, as well as a descriptive analysis of the quantitative variables, offering the mean ± standard deviation or the median (Q1; Q3) values, depending on whether the distribution of the variables was normal or not, respectively.

If distribution was normal, the Student t-test for independent samples was used for the intergroup comparisons of the dependent pre-intervention variables. The Mann-Whitney U test was used for these comparisons if the distribution was not normal. To compare the intervention effectiveness between the groups, we calculated the improvement indexes of the dependent variables using the difference of the final measurement values minus the baseline measurement values. If the distribution was normal, the improvement indexes were compared using the Student t-test for independent samples; if not, the Mann-Whitney U test was used. The effect size was calculated using Cohen’s d.

Correlation between the improvement index of the CVAI and the VAS reflecting the parental perception of head shape change in the entire sample was analyzed. To do so, the Pearson correlation coefficient was calculated when the variables followed a normal distribution. If the distribution of a variable was not normal, the Spearman Rho coefficient was used.

A confidence interval of 95% was established for the analyses. Statistical significance was set at *p* < 0.05. The statistical study was performed following the principles of intention-to-treat analysis, without attributing values in the second assessment to the subjects lost to study throughout the intervention.

## Results

### Study population

**Fig. 2:**
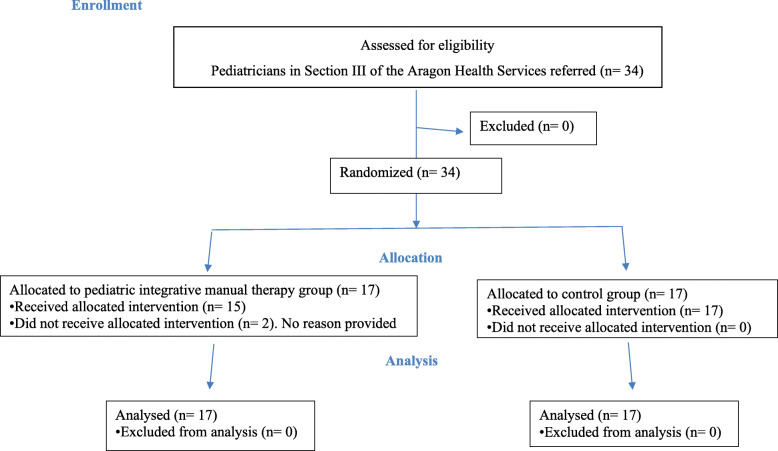
Consolidated Standards of Reporting Trial (CONSORT) flow diagram

A total of 34 subjects were included in the study.Seventeen were assigned to the PIMT group and 17 to the control group (solely caregiver education program). Two subjects were withdrawn from the intervention group, so the final measurement covered 15 subjects in the intervention group and 17 in the control group (Fig. 2). Demographic characteristics were comparable in the 2 groups (Table 1). Anthropometric measurements and head shape were comparable in both groups (Table 2). There were no adverse events with the treatments performed in the study.

**Table 1:** A comparative descriptive analysis of the qualitative variables in the baseline examination.

Features at baseline examination			
Qualitative variables	PIMT Group (*n* = 17)	Control Group (*n* = 17)	*p* value
Sex ^a^			
Female	52.9%	41.2%	0.492
Male	47.1%	58.8%	
Prematurity ^a^	29.4%	5.9%	0.175
Instrumental delivery ^b^	17.6%	23.5%	1.000
Firstborn ^a^	70.6%	70.6%	1.000
Multiple birth ^b^	23.5%	17.6%	1.000
Head position preference ^b^	100%	88.2%	0.485
Pediatrician diagnosis of congenital torticollis ^b^	5,9%	11,8%	1.000
Plagiocephaly side ^a^			
Right	52.9%	76.5%	0.151
Left	47.1%	23.5%	
Transport type ^b^			
Pushchair	100%	94.1%	1.000
Babies backpack	0%	5.9%	

*PIMT* (Pediatric Integrative Manual Therapy) ^a^Statistical analysis using the Chi Square test; ^b^Statistical analysis performed with the Fisher exact test

**Table 2:** Table describing the homogeneity of the quantitative variables in the baseline examination.

Features at baseline examination			
Quantitative variables	PIMT Group (*n* = 17)	Control Group (*n* = 17)	*p* value
Age ^a^ (weeks)	17.29 ± 4.27	17.18 ± 4.55	0.938
Birth weight ^a^ (gr)	3040 ± 605.3	3188 ± 483.7	0.437
Time in prone position with 1 month ^b^ (min)	1 (0; 5)	5 (5; 16)	0.520
Time in prone position with 2 months ^b^ (min)	2 (0.5; 10)	10 (5; 11)	0.228
MCC ^a^ (cm)	40.76 ± 2.01	41.08 ± 2.14	0.610
Cranial Length ^b^ (mm)	131 (129.9; 136.8)	134.8 ± 8.06	0.480
Cranial Width ^a^ (mm)	116.8 ± 8.02	117.1 ± 8.66	0.846
Long diagonal cranial diameter ^a^ (mm)	134.52 ± 7.41	135.39 ± 7.11	0.835
Short diagonal cranial diameter ^a^ (mm)	126.01 ± 7.11	127.35 ± 7.76	0.479
CVA ^b^ (mm)	8.20 (6.50; 11.75)	7 (5.83; 9.33)	0.196
CI ^a^ (%)	88.35 ± 6.39	87.04 ± 7.14	0.522
CVAI ^b^ (%)	6.59 (5.20; 9.30)	5.37 (4.51; 7.86)	0.153

*PIMT* (Pediatric Integrative Manual Therapy). *MCC* (Maximal Cranial Circumference). *CVA* (Cranial Vault Asymmetry). *CI* (Cranial Index). *CVAI* (Cranial Vault Asymmetry Index). ^a^ Statistical analysis using the Student t-test. ^b^ Statistical analysis using the Mann-Whitney U test

### Outcome

The differences between final evaluation and baseline of the anthropometric measurements in the 2 groups are shown in Table 3. The PIMT group showed a statistically significant increase in MCC (2.16 ± 0.69 cm) compared with the control group (1.35 ± 0.75 cm) (*p* = 0.004). Likewise, there was a significant increase in cranial length in the PIMT group (7.57 ± 2.33 cm) in contrast with the control group (4.25 ± 2.47 cm) (*p* = 0.001). CVA presented a significantly greater reduction in the PIMT group (− 4.39 ± 1.51 mm) compared with the control group (− 0.11 ± 2,14 mm) (*p* = 0.000). Our primary outcome, CVAI decreased more in the PIMT group (− 3.72 ± 1.40%) in contrast with the control group (− 0.34 ± 1.72%) (p = 0.000). The CI did not present any statistically significant difference between groups.

**Table 3:** Table summarizing the variables with descriptive and comparative data on their differences between final values-baseline values.

Descriptive and comparative of the differences between final values-baseline values				
Variables	PIMT Group *n* = 15	Control Group *n* = 17	Sig.	Cohen’s d effect size
MCC ^a^ (cm)	2.16 ± 0.69	1.35 ± 0.75	0.004*	1.12
Cranial Length ^a^ (mm)	7.57 ± 2.33	4.25 ± 2.47	0.001*	1.39
Cranial Width ^a^ (mm)	5.42 ± 4.24	3.97 ± 3.11	0.277	0.39
Long diagonal cranial diameter ^b^ (mm)	5.33 (2.33; 6.50)	4.93 ± 2.58	0.610	0.23
Short diagonal cranial diameter ^a^ (mm)	8.88 ± 3.27	5.04 ± 2.71	0.001*	1.28
CVA ^a^ (mm)	−4.39 ± 1.51	−0.11 ± 2.14	0.000*	2.32
CI ^a^ (%)	−0.85 ± 3.63	−0.16 ± 2.00	0.516	0.24
CVAI ^a^ (%)	−3.72 ± 1.40	−0.34 ± 1.72	0.000*	2.16

*PIMT* (Pediatric Integrative Manual Therapy). *MCC* (Maximal Cranial Circumference). *CVA* (Cranial Vault Asymmetry). *CI* (Cranial Index). *CVAI* (Cranial Vault Asymmetry Index). ^a^ Statistical analysis using the Student t-test; ^b^ Statistical analysis using the Mann-Whitney U test; * Significant *p* value

In the VAS for parental perception of head shape change, the parents of the PIMT group subjects evaluated the change perceived in 6.66 ± 2.07 cm (between − 10 cm and + 10 cm). However, the parents of the control group evaluated the change perceived in 4.25 ± 2.31 cm (*p* = 0.004).

In the correlation study, the outcomes showed a statistically significant association (Pearson correlation coefficient = − 0.365; *p* = 0.04) in the entire sample. This association was present between the reduction in CVAI (− 1.92% ± 2.31) and the VAS of head shape change perception (5.38 cm ± 2.49), in the entire sample.

## Discussion

In our study, the use of PIMT has been shown to be more effective than solely applying a caregiver education program. The addition of PIMT to a caregiver education program have produced better outcomes in cranial anthropometric values (increased MCC, cranial length and short diagonal cranial diameter and decreased CVA and CVAI) and in parental perception of head shape changes.

The mean gain in MCC, in the 10-week study period, was 2.16 ± 0.69 cm in the PIMT group and 1.35 ± 0.75 cm in the control group. The growth rates observed by Martini et al. (2018) in healthy babies from 4 months to 12 months was an increase of 3.5 ± 8 cm [29]. For Meyer-Marcoti et al. (2018), the total increase of cranial circumference during the first year was 11–13 cm [56]. In their study on a sample of 40 subjects without cranial asymmetry, a mean MCC of 41.38 cm is observed at 4 months, evolving to 43.23 cm at 6 months (1.85 cm of increase). In our 10-week study period, the natural skull growth in both groups is close to physiological values but it is higher in the PIMT group. Consequently, the MCC skull growth data in the PIMT group were closer to the physiological evolution of the skull of a healthy baby in the same time period [56]. The increase in cranial length was also greater in the PIMT group.

The CVA is one of the main cranial indicators to analyze the possible effectiveness of the PIMT protocol used in this population. Kim et al. (2013) found a significant improvement in this parameter using an orthotic treatment. The variable changed from a mean of 13.28 mm ± 3.57 to 6.48 mm ± 1.92 (− 6.8 mm). The control group in this study changed its values to a lesser degree, going from 11.38 mm ± 3.30 to 10.05 mm ± 1.43 (− 1.33 mm) [29]. In our study, the PIMT treatment (with a CVA change of − 4.39 ± 1.51 mm) was significantly better than the control group (− 0.11 ± 2.14) (*p* = 0.000). The PIMT treatment showed an effectiveness quite similar to orthotic treatments, which are evaluated as the most effective in the literature [29, 57]. Lessard et al. (2011), in a pilot study without a control group on 12 subjects, using a manual intervention, found a significant improvement of − 4.1 mm in the diameter difference [38]. This is close to the outcome in our study.

The cranial index improved in both groups of our study, with no significant differences between them. The predominantly brachiocephalic skull (mainly wide and short) changed towards normal values, without dropping below the limit of 85% that marks the upper limit of the range of normality [24]. In the PIMT group, the CI changed from 88.35 to 87.73%. This was a slightly better improvement than in the control group, which changed from 87.04 to 86.89%. This improvement may be related to the significant improvement in cranial length, a factor needed to balance the CI downwards.

The CVAI improvement was significantly greater in the PIMT group (− 3.72% ± 1.40) than in the control group (− 0.34% ± 1.72) (p = 0.000), with a high size effect (d = 2.16). This variable is the main cranial indicator to analyze the possible effectiveness of the PIMT protocol used in this population. Kim et al. (2013), with a sample of 27 subjects, found a comparable (although somewhat higher) improvement using an orthotic helmet treatment. In this study, the intervention group reduced the CVAI a mean of 5.5%, which was a significant improvement compared with the control group, with a mean reduction of 1.53% [29]. Kluba et al. (2014), with an orthotic helmet intervention, obtained a CVAI reduction of 4.1%, on 128 subjects [57], very close to what we obtained in our study. The studies by di Chiara et al. (2019) [39], Cabrera-Martos et al. (2016) [37] and Lessard et al. (2011) [38], can be considered more similar to our study due to the similarities of the intervention protocol. However, the outcomes in these studies are not comparable with our results because either the CVAI is not measured, or it does not appear with numerical values but with the outcome given in a CVAI severity classification.

Parental perception of head shape change has been one of the variables seen in the literature to assess parents´ satisfaction with the intervention. Naidoo et al. (2015) found that parents whose babies had received helmet treatment were more satisfied than parents whose babies were only indicated repositioning [58]. In our study, the parental perception of improvement in the head shape was better with PIMT in contrast with solely the caregiver education program. In addition, the parental perception of such head shape improvement is related to the CVAI changes in the entire sample.

Our study findings are subject to limitations. The lack of assessments of the skull base asymmetry and of the face constitutes one of them. Including an evaluation of ear position, using a tragus-subnasal measurement with the methodology of Lessard et al. (2011) [38] or Kim et al. (2010) [29], would have made it possible to analyze the change in skull base asymmetry and not just the vault change. Measuring the jaw asymmetry would have increased the evidence available on facial morphology changes obtained using the treatment. Fenton et al. (2018) found a jaw asymmetry in 10% of their sample of subjects with congenital torticollis and PP showing a significant change after 4 months of treatment with physiotherapy [59]. A more exhaustive assessment including facial and skull base measurements is a possible future line of research. It would increase the evidence on the effect of treatments with pediatric manual therapy on cranial base symmetry and on face morphology.

As this study was not a blinded trial, parental perception may have been influenced by the modality of the assigned intervention.

The current lack of a long-term follow-up of our subjects is another limitation. The authors plan to study this in future, by obtaining follow-up data in this research study.

## Conclusions

Manual therapy plus a caregiver education program, in a sample of infants with PP, improved CVAI and led to parental satisfaction more effectively than solely a caregiver education program.

## Data Availability

The datasets used and/or analysed during the current study are available from the corresponding author on reasonable request. All data generated or analysed during this study are included in this published article.

## Abbreviations

CI: Cranial index
CVA: Cranial vault asymmetry
CVAI: Cranial vault asymmetry index
MCC: Maximal cranial circumference
PIMT: Pediatric integrative manual therapy
PP: Positional plagiocephaly
VAS: Visual analogue scale
